# Children’s Exploration of Their Surrogacy Origins in Gay Two-Father Families: Longitudinal Associations With Child Attachment Security and Parental Scaffolding During Discussions About Conception

**DOI:** 10.3389/fpsyg.2020.00112

**Published:** 2020-01-31

**Authors:** Nicola Carone, Lavinia Barone, Demetria Manzi, Roberto Baiocco, Vittorio Lingiardi, Kathryn Kerns

**Affiliations:** ^1^Department of Brain and Behavioral Sciences, Lab Attachment and Parenting-LAG, University of Pavia, Pavia, Italy; ^2^Department of Developmental and Social Psychology, Sapienza University of Rome, Rome, Italy; ^3^Department of Dynamic and Clinical Psychology, Sapienza University of Rome, Rome, Italy; ^4^Department of Psychological Sciences, Kent State University, Kent, OH, United States

**Keywords:** gay father family, surrogacy origins, attachment security, parental scaffolding, middle childhood

## Abstract

Evidence is lacking about the factors that are pivotal in enhancing the exploration of surrogacy origins in children of gay fathers during middle childhood. The present study examined the separate and combined influences of child attachment security and parental scaffolding (i.e., fathers’ attempts to accept, encourage, and emotionally support their children’s expression of thoughts and feelings) during discussions about conception on children’s exploration of their surrogacy origins in 30 Italian children born to gay fathers through gestational surrogacy. Within each family, both father–child dyads (*n* = 60) participated in a 5-minute videotaped conversation regarding an aspect of the child’s conception when children were mean aged 8.3 years (t1). At this time, children were also administered the Security Scale Questionnaire to evaluate their attachment security. Approximately 18 months later (t2; *M*_age_ = 9.9 years), children were interviewed about their surrogacy origins. Linear mixed models (LMMs) for longitudinal data indicated that, with higher levels of parental scaffolding, only children who perceived greater attachment security reported greater exploration of their surrogacy origins. The findings are the first to underscore the importance of conversations about surrogacy within the context of parent–child attachment relationships, as well as the importance of fathers sensitively supporting their children as they explore their origins during middle childhood. In doing so, it is expected that fathers will likely facilitate their children’s positive integration of their surrogacy conception into a coherent sense of identity during adolescence.

## Introduction

An increasing number of gay men are having children via surrogacy (Norton et al., 2013; Blake et al., 2017; Carneiro et al., 2017)—a practice by which a woman (the “surrogate”) bears a child for the intended parent(s). Two types of surrogacy are possible: (1) *genetic* surrogacy, in which conception uses the sperm of one of the intended fathers and the egg of the surrogate, who carries the child to term; and (2) *gestational* surrogacy, in which the surrogate has no genetic relationship to the child and fathers select an egg donor with whom they might have contact in the future (an open-identity donor) or one of whom they have no identifiable information (an anonymous donor)—although the possibility of achieving complete anonymity is in doubt (Harper et al., 2016). In Italy, where the present study was conducted—and similar to many other European countries (e.g., Spain, France, Sweden, Denmark)—both forms of surrogacy (i.e., genetic and gestational) are illegal; thus, those who wish to use surrogacy to conceive must do so transnationally (e.g., in California or Canada) (Carone et al., 2017; Sydsjö et al., 2019).

Similar to disclosure in lesbian and single parent families (Tallandini et al., 2016; Faccio et al., 2019), disclosure of a child’s surrogacy origins in gay two-father families is thought to be relatively straightforward and to occur earlier than in heterosexual two-parent families, likely due to the visible absence of a mother and the child being raised by two fathers. However, to date, to the best of our knowledge, only two studies have investigated the manner in which disclosure of surrogacy conception occurs in this family type (Blake et al., 2016; Carone et al., 2018a). These studies found that almost all children were told (to different degrees) before the age of four about the involvement of a woman who carried them in her belly, though more sophisticated aspects related to the conception (e.g., the presence of another woman who donated an egg or the identity of the father who used his sperm to conceive) tended to be disclosed only when the children were older. Despite the significant contribution of these studies, however, evidence is lacking about the factors that are pivotal in enhancing exploration of surrogacy origins in children of gay fathers during middle childhood.

An investigation of this topic may be particularly appropriate when children are in middle childhood (aged 6–12 years), because, by the age of 6–8 years, children begin to grasp the significance of the biological concept of family and the implications of a lack of biological connections among family members (Solomon et al., 1996; Williams and Smith, 2010). For children born to gay fathers through surrogacy, such knowledge may raise questions about the nature of their family relationships (e.g., “Who is part of my family?”) and the role played by the surrogate and egg donor in their family arrangement (e.g., “Who am I genetically related to?” and “Whose body did I grow in?”). This pairs with the fact that, in middle childhood, children develop their social perspective-taking abilities and acquire new coping strategies, making them more capable of processing potentially stressful experiences (Compas et al., 2001).

At the beginning of middle childhood, in fact, children transition to primary school. For children of gay fathers, this transition may increase the likelihood that they will be confronted with family types that largely differ from theirs (e.g., heterosexual two-parent families through spontaneous conception) and this may lead them to examine what their family form means to them and to others. They may also be questioned by peers on the uniqueness of their family composition, in terms of both the absence of a mother and their conception through surrogacy. In this context, gay two-father families face a double task: fathers must create an emotional atmosphere for their children to safely explore what surrogacy means to them and the implications of such conception; and children must have a family environment in which they are able to safely ask questions about their surrogacy conception whilst continuing to feel emotionally supported by their fathers.

In this regard, research with adoptive families (Wrobel et al., 1996, 2003; Brodzinsky, 2006; Skinner-Drawz et al., 2011; Farr et al., 2014) and assisted reproduction families (MacDougall et al., 2007; Isaksson et al., 2012; Tallandini et al., 2016; Van Parys et al., 2016a, b) has largely documented that communication about conception is a core task of families that have not been formed through spontaneous conception. Often, this communication is not a one-time event, but a dynamic process between parents and children that varies in intensity as children mature (Brodzinsky, 2005, 2006; MacDougall et al., 2007). Throughout this process, parental attitudes toward their children’s conception may be even more important than the information disclosed or the frequency with which the subject is raised (Wrobel et al., 2003; Van Parys et al., 2016a, b).

One aspect which accounts for parental attitudes toward child’s conception is *parental scaffolding*. Specifically, consistent with Leibowitz et al. (2002) definition, in the context of this study, parental scaffolding refers to parents’ acceptance, encouragement, and emotional support of their children’s expression of feelings about their surrogacy origins during discussions about conception. Translating both the findings of prior research (Wrobel et al., 2003; Van Parys et al., 2016a, b) and the idea of parental scaffolding in the context of gay two-father surrogacy families suggests that an open and sensitive exchange of surrogacy-related information and support of children’s thoughts and feelings about their surrogacy conception should facilitate children’s exploration of their unique origins. In this vein, one could expect that, when fathers facilitate open emotional discussions with their children and adapt to their changing needs for communication about surrogacy, the children are more likely to have positive feelings about their conception and feel free to explore their surrogacy origins. This prediction is supported by the literature on adoption (Grotevant, 1997; Kohler et al., 2002; Brodzinsky, 2005; Neil, 2012; Farr et al., 2014).

In addition to parental scaffolding during discussions about conception, from the perspective of attachment theory (Bowlby, 1988) the extent to which children feel free to explore their origins is also likely intertwined with their perceived attachment security to their fathers. The *secure base phenomenon* (Bowlby, 1988) is one of the key tenets of attachment theory and defines the purposeful balance between children’s use of their parents as both a secure base from which to explore and learn about their surroundings and a safe haven to return to if a threat arises or fatigue or illness hits. In a similar vein, Grossmann et al. (2008) introduced the companion idea of *secure exploration* to refer to “a child’s ability to organize emotions and behaviors open-mindedly, non-defensively, and with concentration when responding to “curious” events, and to do so with care; and the child’s confidence in an attachment availability and helpfulness, should help be needed” (p. 859). Both factors are then based on “attachment figure’s observable sensitivity and support during distressing situations, when the child’s attachment system or need to explore is aroused” (p. 859). In middle childhood—a period in which children begin to balance separateness from and connectedness to their parents (Bosmans and Kerns, 2015)—children may perceive any exploration of their surrogacy origins (e.g., exploring their thoughts and feelings toward the surrogate and/or egg donor; initiating conversations about their genetic origins and/or family structure) as threatening and intimidating, because it is new and unfamiliar, and because they do not know how their fathers will react to their curiosity (Lingiardi and Carone, 2019).

Preliminary indications that attachment theory is relevant for understanding children’s experiences of their origins in families formed by assisted reproduction stem from two studies conducted with children of lesbian and single mothers through donor insemination, during middle childhood (Zadeh et al., 2017) and adolescence (Slutsky et al., 2016). The findings of these studies showed that, across these developmental periods, donor-conceived children who reported secure-autonomous attachment to their mothers were more curious about their conception and felt more positive regarding their donor. However, both studies tested a linear association between attachment patterns and children’s exploration of their origins, and did not include parents’ own experiences of the assisted conception as, for example, parental scaffolding during discussions about conception. Specifically, the combined consideration of child attachment security and parental scaffolding seems crucial, as prior research with lesbian and single mother families through donor insemination have indicated that parents likely operate as “gatekeepers” who negotiate children’s relationship with their donor (Hertz, 2002). Furthermore, loyalty toward parents (especially the non-biological parent) may inhibit children from seeking information and expressing curiosity about their donor (Vanfraussen et al., 2003), and both parents and children may report discrepancies in the meaning and significance they attribute to the donor (Tasker and Granville, 2011).

By this perspective, individual variations in children’s explorations of their surrogacy origins may be best explained by considering how discussions about conception occur within the family and the extent to which the children feel secure in their attachment relationships with their fathers. Furthermore, these factors should be considered in conjunction, rather than separately. To this aim, the present study investigated the following research question: Does child attachment security longitudinally moderate the influence of parental scaffolding during discussions about conception on children’s exploration of their surrogacy origins? It was expected that, when fathers were emotionally supportive and encouraged their child’s expression of feelings and questions related to conception, children who reported greater attachment security to their fathers would be more likely to explore (i.e., to express interest in receiving more information/to show serious, reflective, or meaningful thinking about) their surrogacy origins than children with less secure attachment relationships.

## Materials and Methods

### Participants

The sample comprised 30 children born through gestational surrogacy abroad and their 66 gay fathers. At time 1 (t1), children were mean aged 8.3 years (*SD* = 1.8; age range: 6–12 years), whereas at time 2 (t2; approximately 18 months later), children’s mean age was 9.9 years (*SD* = 1.8; age range: 7.5–13.5 years). In families with more than one child in the relevant age range, the oldest child was studied. At t1, families were recruited in the context of a larger, in-depth study of child adjustment and parenting in gay father surrogacy families (Carone et al., 2018b, 2019). Multiple strategies were used to include as diverse a sample as possible, through the main Italian association of same-sex parents (*n* = 14, 46.7%), same-sex parent Internet groups and forums (*n* = 7, 23.3%), events at which same-sex parents were in attendance (*n* = 3, 10.0%), and snowballing (*n* = 6, 20.0%). The inclusion criteria for gay father families were that the couple had lived together since the child’s birth, resided in Italy, and had conceived through surrogacy. Table 1 presents socio-demographic details on the sample.

**TABLE 1:** Descriptive statistics of socio-demographic information (*n* = 30 families).

	**Gay two-father families (*n* = 30)**
**Child sex (male)**	14 (46.7%)
**Number of siblings**	
0	10 (33.3%)
1	18 (60.0%)
2 or more	2 (6.7%)
**Father ethnicity (Caucasian)**	58 (96.7%)
**Family residence**	
Northern Italy	14 (46.7%)
Central Italy	15 (50.0%)
Southern Italy	1 (3.3%)
**Father education (bachelor’s degree or higher)**	49 (81.7%)
**Father occupation (professional/managerial)**	50 (83.3%)
**Father work status (full-time)**	60 (100%)
**Length of couple relationship**	
<10 years	8 (26.7%)
11–15 years	7 (23.3%)
>15 years	15 (50.0%)
	*M (SD)*
**Child age at t1 (months)**	99.70 (20.01)
**Child age at t2 (months)**	117.87 (20.10)
**Father age (years)**	46.55 (6.61)
**Annual household income**	120,433.33 (55,138.66)

*Where not otherwise specified, all information refers to t2. For the individual parent variables of ethnicity, education, occupation, work status, and age, the total *n* is 60, rather than 30.*

### Procedure

Three researchers at t1 and one researcher at t2 visited families at home and administered the study measures (i.e., questionnaires, interviews, and observational tasks) to both fathers and children; all researchers had been trained in the study techniques. Study approval was obtained from the Ethics Committees of the Department of Developmental and Social Psychology, Sapienza University of Rome (at t1; Protocol Number: 4 VII/16), and the Department of Brain and Behavioral Sciences, University of Pavia (at t2; Protocol Number: 033/19). Written informed consent was obtained from all fathers, who also gave consent for their children to participate. Children gave verbal assent. All participants were reminded that their responses would be confidential and that participation in all or part of the study could be terminated at any time; such information was conveyed to the children in an age appropriate manner, both prior to and during the data collection. Of relevance, data for three children who took part in phase 1 of the study on their exploration of their surrogacy origins was not collected at t2 because their parents did not consent.

### Measures

#### Child Attachment Security (at t1)

Children were administered the 21-item version of the Security Scale Questionnaire (Kerns et al., 2015; see also Carone et al., 2019) to assess their perceived attachment security to their fathers. In order to ensure that the youngest children (aged 6–7 years) understood the questions, each item was read aloud to them. Harter’s (1982) “Some kids… Other kids…” format was used in administering this scale twice (one for each father) to each child in order to assess their safe haven (e.g., “Some kids feel their dad really understands them BUT Other kids feel like their dad really does not understand them”) and secure base support constructs (e.g., “Some kids think their dad encourages them to be themselves BUT Other kids do not think their dad encourages them to be themselves”)—which, together, define the secure base phenomenon (Bowlby, 1988). For each question, children indicated which statement was more characteristic of them and indicated whether the statement was *really true* (1) or *sort of true* (4) for them. In addition to generating two item scores (i.e., a safe haven score and a secure base score) for each parent, the scale also generates a total score of attachment security for each parent by averaging the item scores. Higher scores indicate higher levels of children’s perceived attachment security. In the present study, only the total attachment security score for each father was used. The reliability and validity of the SS have been assessed in both child and adolescent samples, showing moderate stability over time and convergence with observations of children’s interactions with their parents (Brumariu et al., 2018). In the present study, Cronbach’s alpha was 0.78.

#### Parental Scaffolding During Surrogacy-Related Discussions (at t1)

Each father–child dyad was instructed to have a 5-minute conversation about an aspect of their child’s surrogacy conception, with the researcher out of the room (he or she returned to the room once the 5 min had elapsed). The father–child dyads were not given any guidelines regarding a specific aspect of the surrogacy to discuss or how they should choose this aspect, because the manner in which they decided on an aspect was considered indicative of their emotional openness (e.g., it was considered relevant if the child brought up an aspect and the father dismissed its significance or refused to talk about it); it was also thought that fathers’ approaches to choosing an aspect to discuss would demonstrate meaningful parent–child differences in the discussion of emotionally charged events (Fivush, 1991). All 5-minute conversations were videotaped and later coded on both individual (i.e., parental scaffolding and children’s emotional openness to discussing their feelings about their conception) and dyadic (i.e., the quality of the parent–child emotional conversation about the conception) dimensions. Only the individual coding of *parental scaffolding* (i.e., fathers’ attempts to accept, encourage, and provide emotional support for their child’s expression of feelings related to conception) was used, and this was rated on a 5-point Likert scale, with 1 describing fathers who simply noted the event without discussing it or engaged in an extremely short discussion without expressing any emotional support; and 5 describing fathers who asked their child to expand on his or her thoughts and feelings and helped the child to respond, and/or fathers who clearly acknowledged and encouraged their child to express his or her thoughts and feelings by validating and paraphrasing them, and/or fathers who elaborated on the emotional component of an event related to their child’s conception. Scoring used the criteria indicated by Gentzler et al. (2005) and Leibowitz et al. (2002) for coding parent–child emotional communication. A second coder, blind to participant data, rated 30% of the interactions (*n* = 18); this resulted in an interrater reliability of κ = 0.79.

#### Children’s Exploration of Their Surrogacy Origins (at t2)

Children were asked questions about their surrogacy conception information gap, including: “What more would you like to know about your surrogacy conception?” and “What information would you like?”. Follow-up probes were used to determine the intensity of children’s curiosity about the identified content. This interview format was adapted from the Minnesota/Texas Adoption Project (Grotevant and McRoy, 1997; Wrobel et al., 2013). The extent to which children were interested in and/or curious about their conception (shown, e.g., by questions about the surrogacy procedure or the egg donor’s motivation, or by particular feelings expressed toward the surrogate) was considered an indicator of exploration of their surrogacy origins and was coded using a 4-point scale, on which (1) indicated children who expressed no interest in receiving additional information or children who showed no serious, reflective, or meaningful thinking about their surrogacy origins (no/minimal exploration); (2) indicated children who desired new information but claimed that knowing the information would not make a big personal difference to them, as well as children with low interest in the information (low exploration); (3) indicated children who wanted to gain particular information (moderate exploration); and (4) indicated children who stated an intense desire for particular information that was of high importance to them (great exploration). A second coder, blind to participant data, rated 30% of the interviews (*n* = 9); this resulted in an interrater reliability of κ = 0.75.

### Data Analysis

To identify the likelihood that the data would detect the factors that best explained children’s exploration of their surrogacy origins, given a set of parameters (Wagenmakers, 2007; van de Schoot et al., 2014), several linear mixed models (LMMs) for longitudinal data (Goldstein, 1988) were computed and compared. To overcome the possible limitations of the small sample size while maintaining predictive accuracy, LMMs were compared using the total coefficient of determination (TCD) and Bayesian information criterion (BIC) (Schwarz, 1978) methods. The TCD method shows the combined effect of model variables on the dependent variable; the BIC method measures the efficiency of the parameterized model in predicting data and penalizes according to model complexity (i.e., with respect to the number of unnecessary parameters). The higher the TCD (range 0–1), the more variance is explained by the model; the lower the BIC, the better the model fit. Consequently, the model with the highest TCD and lowest BIC can be said to best fit the data. The set of investigated predictors was comprised of parental scaffolding during discussions with their child about their child’s conception, child attachment security, and children’s and fathers’ demographic information (i.e., child age and gender; parents’ age, education, and annual household income), as well as the additive and interactive effects of these variables (with all variables centered in advance, in order to reduce multicollinearity).

To evaluate interactive effects, the Johnson–Neyman technique (Johnson and Neyman, 1936; Preacher et al., 2006) was used to inspect the range of values (i.e., regions of significance) of the moderator for which the independent and dependent variables were significantly associated. This technique was selected over simple slopes analysis because the latter probes significant interactions at two arbitrarily specified moderator levels (i.e., ±1 *SD*), even though it is a continuous dimension without a natural break point (for a wider discussion, see Dearing and Hamilton, 2006). All analyses were performed using the statistical software R (R Development Core Team, 2018), with the lme4 package being used for mixed-effects model, the lmerTest being used for computing the *p*-values of main and interaction effects of the best model selected, and the effects package being used for exploring interaction effects.

## Results

Table 2 shows the associations between children’s attachment security (at t1), parental scaffolding during discussions with their child about their child’s conception (at t1), and children’s exploration of their surrogacy origins (at t2), after controlling for children’s age (at t2).

**TABLE 2:** Mean scores and associations between child attachment security, parental scaffolding during discussions about conception, and children’s exploration of their surrogacy origins, after controlling for child’s age at t2.

	**1**	**2**	**3**	***M***	***SD***	***Observed values [expected values]***
1. Attachment security (t1)	1.00			3.14	0.48	1.95–4.00 [1–4]
2. Parental scaffolding (t1)	0.16	1.00		3.57	1.00	2–5 [1–5]
3. Children’s exploration of their surrogacy origins (t2)	0.42**	0.33**	1.00	2.97	1.00	1–4 [1–4]

t1 = time 1; t2 = time 2, approximately 18 months after t1. In each family, fathers’ scores were averaged. ** *p* < *0.01*.

### Parental Scaffolding During Discussions About Conception and Child Attachment Security as Predictors of Children’s Explorations of Their Surrogacy Origins

Table 3 displays fit indices and model comparisons. Only models with better fit than the null model (intercept only) were reported (i.e., models containing child gender; and parents’ age, educational level, and household annual income were excluded). Model 4, containing children’s age and the main and interactive effects of parental scaffolding and child attachment security as predictors, best explained children’s exploration of their origins with the highest global variance (i.e., TCD = 0.34) and the lowest BIC (163.22). Specifically, greater attachment security, β = 0.30, *p* = 0.009, the interaction between child attachment security and parental scaffolding, β = 0.23, *p* = 0.048, and child age, β = 0.02, *p* < 0.001, predicted greater exploration in children, whereas the main effect of parental scaffolding was marginally significant, β = 0.20, *p* = 0.072.

**TABLE 3:** Linear mixed model comparisons and model fit indices predicting children’s exploration of their surrogacy origins at t2.

**Outcome: Children’s exploration of their surrogacy origins (t2)**			***CI***		
	
	**B (*SE*)**	**β**	**[*25%*–*75%*]**	***TCD***	***BIC***
Model 0 (null model – intercept only)					176.36
Model 1				0.20***	168.96
Parental scaffolding (t1)	0.25 (0.12)	0.25*	[0.02–0.48]		
Child attachment security (t1)	0.76 (0.25)	0.37**	[0.28–1.25]		
Model 2				0.31***	163.41
Child age (t2)	0.02 (0.01)	0.36**	[0.01–0.03]		
Parental scaffolding (t1)	0.25 (0.11)	0.25*	[0.04–0.47]		
Child attachment security (t1)	0.71 (0.23)	0.35**	[0.27–1.16]		
Model 3				0.20**	171.76
Parental scaffolding (t1)	0.22 (0.12)	0.22^†^	[−0.02–0.46]		
Child attachment security (t1)	0.72 (0.26)	0.36*	[0.21–1.21]		
Parental scaffolding * Child attachment security (t1)	0.28 (0.26)	0.14	[−0.22–0.78]		
**Model 4**				0.34***	**163.22**
**Child age (t2)**	0.02 (0.11)	0.39***	[0.01–0.03]		
**Parental scaffolding (t1)**	0.20 (0.11)	0.20^†^	[−0.01–0.41]		
**Child attachment security (t1)**	0.61 (0.22)	0.30**	[0.17–1.05]		
Parental scaffolding * Child attachment security (t1)	0.47 (0.23)	0.23*	[0.01–0.92]		

CI = confidence interval. The emboldened model (i.e., Model 4) is the one that best fit the data, with the highest TCD and lowest BIC. ^†^*p* < 0.08; * *p* < 0.05; ** *p* < 0.01; *** *p* < 0.001.

**FIGURE 1:**
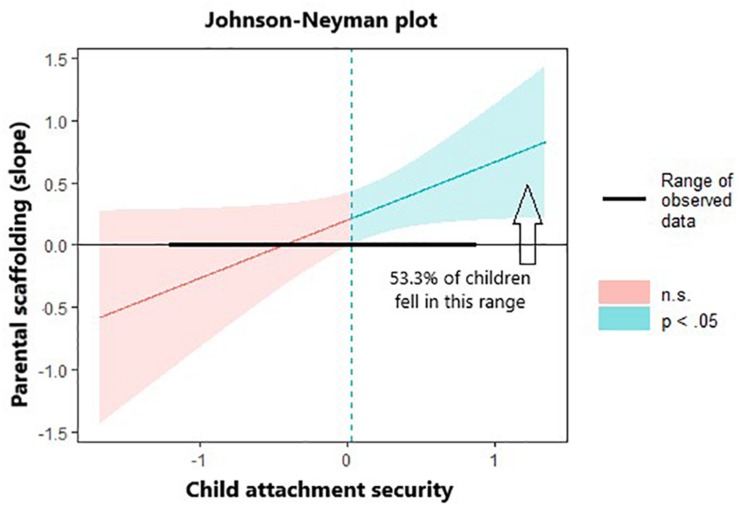
Johnson-Neyman plot.

The follow-up Johnson-Neyman technique identified the region of significance on the centered moderator (i.e., child attachment security) to range from **−**73.32 (lower bound) to 0.03 (upper bound), indicating that any given simple slope outside this range was statistically significant. Given that centered attachment security scores at t2 ranged from −1.19 to 0.86 (range of raw observed scores: 1.95–4.00) and the interactive term was positively associated with the outcome, it may be concluded that, in the presence of higher levels of parental scaffolding, only children who perceived greater attachment security (i.e., mean SS score ≥3.18; approximately 53.3% of children fell in this range) reported greater exploration of their surrogacy origins (for a graphical representation, see Figure 1).

## Discussion

This study was the first investigation of the longitudinal influence of child attachment security and parental scaffolding during parent–child discussions about the child’s conception in predicting children’s exploration of their surrogacy origins in gay two-father families during middle childhood. In line with expectations, in families in which fathers were particularly capable of remaining empathically attuned whilst supporting their children in elaborating upon their questions regarding surrogacy, children expressed their thoughts and feelings toward the surrogate and/or egg donor and initiated conversations about their genetic origins and family structure to a greater extent only when they also reported greater attachment security to their fathers. Said differently, the degree of parental scaffolding observed in fathers during discussions with their children about their surrogacy conception longitudinally predicted children’s greater exploration of their surrogacy origins only in more secure children.

In this vein, the findings contribute to the emerging literature about how individual differences in child attachment security to parents are fundamental in children’s own exploration of their assisted conception (Slutsky et al., 2016; Zadeh et al., 2017), insofar as questions children ask to their fathers or to themselves about the surrogacy procedure (e.g., “I was wondering whether both dad and daddy, or only daddy, put their seed in the [surrogate’s name]’s tummy because daddy and I are blonde, whereas dad is not”) or reflections children make upon different motivations egg donors and surrogates might have in helping their fathers in creating their family (e.g., “I cannot understand why [egg donor’s name] helped us if she then disappeared…”) are a form of exploration facilitated by greater father-child attachment security.

Two considerations—one methodological and one theoretical—may be relevant for interpreting this finding. First, on a methodological level, it should be noted that most fathers were rated as quite open and sensitive in encouraging children to express their thoughts and feelings about surrogacy; furthermore, the number of children who scored at the low to medium end of the attachment security scale was very small (Table 2). Interpreted in the context of the small sample size, this finding suggests that the potential effect of both child attachment insecurity and fathers’ limited scaffolding when children were interested in exploring their origins more deeply may have gone undetected.

Second, on a theoretical level, attachment theory (Bowlby, 1988; Grossmann et al., 2008) provides an in-depth explanation for why children may perceive or even experience any exploration of their surrogacy origins as stressful and generative of uncertain outcomes, as well as why a secure father–child relationship, in combination with high parental scaffolding, may support such an exploration. Children might wonder how their fathers will react to their interest in knowing more about their egg donor or having more frequent contact with their surrogate (Lingiardi and Carone, 2019). The vast geographical distance between gay father families and their surrogate, as well as an egg donor’s anonymity or limited contact between fathers and children with the egg donor (Blake et al., 2016; Carone et al., 2018a) may further contribute to making both the surrogate and the egg donor “unfamiliar” and probably undefined to the child.

In addition, vital to attachment theory (Bowlby, 1980) is the conviction that individuals are guided by prototypes of their earliest relationships (i.e., internal working models), which shape their expectations of self and other and serve as guides for interpreting and managing negative emotions. Salient to the present study, in middle childhood, children who are securely attached to their parents rely on a representation of both fathers as secure bases who consistently support their exploration and safe havens who protect them when their attachment system is activated (e.g., by a threatening situation); however, they also rely on a representation of the self as a person who is comfortable with both intimacy and autonomy (Bosmans and Kerns, 2015). In this perspective, it is perhaps unsurprising that fathers who supported and acknowledged their children in expressing their thoughts and feelings related to their conception had children who were more likely to be engaged in the challenging task of exploring their surrogacy origins when they also perceived greater attachment security.

When interpreting these findings, caution should be exercised for several reasons. First, the small sample size, the convenience nature of the sampling, and the rarefied high socio-economic status of the families restricted the representative nature of the sample. Second, a further aspect of selectivity relates to the ways in which the fathers, themselves, came to terms with their surrogacy conception and the information they disclosed to their children, as they were likely to have relatively high levels of emotional support and an overall positive experience of the surrogacy conception. Third, the limited sample size for each cell prevented a larger investigation of whether children’s exploration of their origins differed due to gender and/or their level of understanding about their surrogacy conception. As the number of gay two-father surrogacy families grows, future studies should address these issues, as there is evidence that, in these families, children’s understanding of and questions about their surrogacy conception (with respect to, e.g., the different roles of the surrogate and the egg donor and the genetic parent–child relationship) may influence parental disclosure (Blake et al., 2016; Carone et al., 2018a). In addition, the results of adoption studies (Neil, 2012) suggest that girls may be more advanced in expressing their feelings toward conception than boys, possibly due to gender-typic emotional socialization by parents (Morris et al., 2007). Whether this finding also applies to gay two-father families, in which extra efforts might be required for fathers to engage in conversation with their son about his feelings related to his surrogacy conception, is worthy of exploration within a larger sample.

Fourth, as the Security Scale does not detect different types of attachment insecurity, the present study was not able to verify for the present sample prior findings with donor-conceived children that preoccupied and dismissing children differ in their experiences of their conception (Slutsky et al., 2016), with insecure-dismissing children being less likely to express curiosity in donor conception. Fifth, children’s participation and concentration during the home visits were quite variable, and thus their understanding of the questions may have reflected their mood on the day. In the same vein, as noted by Van Parys et al. (2016b), it cannot be ruled out that, given the one-to-one interview context with an adult interviewer whom the child had only just met for the first time, the children might have been selective regarding the material they disclosed. Finally, it may be meaningful to consider that the children were being asked about a topic (i.e., their surrogacy conception) that was unlikely to have been discussed in their daily communication (Carone et al., 2018a).

Notwithstanding these limitations, the study presented a number of strengths. The longitudinal design and the attachment framework (Bowlby, 1988; Grossmann et al., 2008) enabled insights from the adoption (Grotevant, 1997; Kohler et al., 2002; Brodzinsky, 2006; Farr et al., 2014) and donor conception literature (Slutsky et al., 2016; Zadeh et al., 2017) to be extended to children born through surrogacy in gay two-father families, who must navigate unique challenges when processing their surrogacy origins (especially from middle childhood onward, when they enter primary school and confront their family diversity on a daily basis). The task of dealing with one’s surrogacy origins may be even more thorny for gay fathers and children living in countries such as Italy, where surrogacy is a highly contentious path to parenthood, same-sex couples have no domestic access to assisted reproduction, and legislation does not recognize the relationship between the non-genetic (non-legal) parent and the child (Lingiardi and Carone, 2016a, b). Further strengths of the study were the inclusion of children’s voices, which are generally underrepresented in studies with assisted conception families, even though children are “full” research participants, rather than objects of research (Mason and Hood, 2011). In addition, use of the extended version of the Security Scale (Kerns et al., 2015; Carone et al., 2019) was particularly valuable, as it covered both components (i.e., safe haven and secure base support) of the secure base phenomenon (Bowlby, 1988), which characterizes parent–child attachment in middle childhood (Bosmans and Kerns, 2015).

Prior to this study, it was not known how discussions about surrogacy conception in gay two-father families relate to parents’ own experiences of the assisted conception and children’s attachment relationships with their fathers. The present study is thus particularly informative, because the rationale for disclosing to one’s child his or her surrogacy origins and the choice of what to disclose is never straightforward, given that it touches upon the meaning of social and genetic ties (Haimes and Weiner, 2000). Through the lens of attachment theory (Bowlby, 1988; Grossmann et al., 2008), it may be said that the quality of the parent–child attachment relationship is crucial in helping children freely and safely explore the unfamiliar topic of their conception, especially when this may be perceived as a threatening and uncomfortable process.

By the same token, insofar as mental health professionals and relevant scientific societies (e.g., Ethics Committee of the American Society for Reproductive Medicine, 2018) are increasingly encouraging the disclosure of assisted conception, the findings underscore the importance of conversations about surrogacy within the context of parent–child attachment relationships, as well as fathers’ sensitive support for their child’s exploration of his or her origins. To a wider extent, the findings also suggest that fathers should be prepared to talk with their children about their surrogacy conception, as children’s need for information likely change over the life course; and fathers should also respect their children’s curiosity toward aspects related to their story, break down barriers to information, and, in so doing, prevent future adjustment problems. Consistent with prior studies in the fields of adoption (Grotevant, 1997) and donor insemination (Slutsky et al., 2016), it is thus expected that fathers’ enhancement of their children’s secure exploration of their origins in middle childhood will facilitate children’s positive integration of surrogacy conception into a coherent sense of identity during adolescence.

## Data Availability Statement

The datasets generated for this study are available upon reasonable request to the corresponding author.

## Ethics Statement

The studies involving human participants were reviewed and approved by the Ethics Committee of the Department of Developmental and Social Psychology, Sapienza University of Rome; Ethics Committee of the Department of Brain and Behavioral Sciences, University of Pavia. Written informed consent to participate in this study was provided by the participants’ legal guardian/next of kin.

## Author Contributions

NC conceived and designed the study, collected data at time 1 and time 2, performed the statistical analysis, and drafted the manuscript. LB and KK contributed in the interpretation of the results. DM collected data at time 1, organized the database, coded data, and assisted in data analysis. RB and VL supervised the first phase of the project. All authors contributed to manuscript revision, read and approved the submitted version.

## Conflict of Interest

The authors declare that the research was conducted in the absence of any commercial or financial relationships that could be construed as a potential conflict of interest.
